# RMI2 plays crucial roles in growth and metastasis of lung cancer

**DOI:** 10.1038/s41392-020-00295-4

**Published:** 2020-09-03

**Authors:** Weixiang Zhan, Yina Liu, Ying Gao, Run Gong, Wen Wang, Ruhua Zhang, Yuanzhong Wu, Tiebang Kang, Denghui Wei

**Affiliations:** 1 State Key Laboratory of Oncology in South China, Collaborative Innovation Center for Cancer Medicine, Sun Yat-sen University Cancer Center, 510060 Guangzhou, China; 2 The Sixth Affiliated Hospital, Sun Yat-sen University, 510000 Guangzhou, China; 3 Department of Abdominal Oncology, The Cancer Center of the Fifth Affiliated Hospital of Sun Yat-sen University, 519000 Zhuhai, Guangdong China

**Keywords:** Lung cancer, Metastasis, Oncogenes

**Dear Editor**,

Lung cancer is the most commonly diagnosed cancer and the leading cause of cancer death in the world, but its therapeutic targets are still being explored. Genome instability as a key hallmark of cancer not only contributes to cancer initiation and progression,^1^ but also creates vulnerabilities that are relatively specific to cancer cells, which may be potential therapeutic targets for cancer patients. During DNA Double-Strand Breaks (DSBs) repair, BTR (BLM-Topo IIIα-RMI1/RMI2) complex promotes the dissolution of double Holliday junctions to form non-crossover products and is often considered as a tumor suppressor.^2^ However, the function of each individual component of this BTR complex in cancer remains largely unknown.

Using the GEPIA tool based on TCGA and GTEx databases, we found that the mRNA levels of BLM, RMI1, and RMI2 were simultaneously increased in multiple cancer types, while TOP3A was not altered in the tested cancer types. Notably, RMI2, but neither BLM nor RMI1, was up-regulated in Adrenocortical carcinoma (ACC), Kidney Chromophobe (KICH), Liver hepatocellular carcinoma (LIHC), Lung adenocarcinoma (LUAD) and Thyroid carcinoma (THCA) (Supplementary Fig. S1a). Furthermore, the mRNA levels of RMI2 were up-regulated in other cancer types (21 out of 33 cancer types), including breast invasive carcinoma (BRCA), ovarian serous cystadenocarcinoma (OV), pancreatic adenocarcinoma (PAAD), uterine Corpus Endometrial Carcinoma (UCEC) and so on. Kaplan–Meier plotter revealed that high mRNA levels of RMI2 were correlated with poor prognoses in BRCA, LIHC, LUAD, OV, PAAD, UCEC (Supplementary Fig. S1b, c). Moreover, using a LUAD tumor tissue microarray, RMI2 was also higher by immunohistochemistry (IHC) in lung tumor tissues compared to normal lung tissues, and high RMI2 protein levels were significantly associated with poor outcomes in LUAD patients (Fig. 1a, b).

**Fig. 1:**
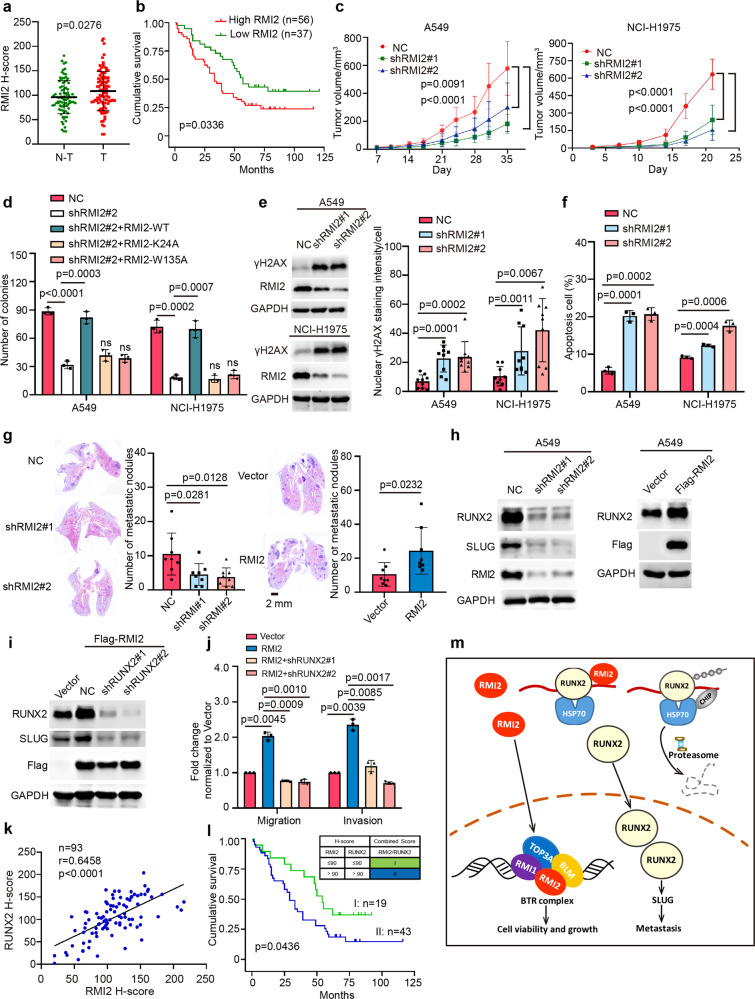
RMI2 plays crucial roles in growth and metastasis of lung cancer. **a** IHC staining of the primary human LUAD tissue microarray and adjacent noncancerous tissues. Scatter plot graph showing a statistical analysis of RMI2 expression in LUAD and adjacent noncancerous tissues. Data are means ± s.e.m. *p* = 0.0276 by student’s *t*-test. **b** Overall survival curves were generated based on the protein levels of RMI2 in LUAD tissue microarray. *p* = 0.0336 using Kaplan–Meier plots and compared with the log-rank test. **c** The indicated stable cells were xenografted subcutaneously on the flank of nude mice (*n* = 8/group). Visible tumors were measured twice a week. Data are means ± s.e.m. of tumor volume. *p* values were calculated by two-way ANOVA. **d** The indicated stable cells were subjected to colony formation assay. The bars indicate the s.e.m. The results are expressed as the mean ± s.e.m. (*n* = 3). *p* values were calculated by student’s *t*-test. n.s no significance. **e** The indicated stable cells were analyzed by western blotting and Nuclear γH2AX staining intensities per cell were quantified. Data are means ± s.e.m. *p* value was calculated by student’s *t*-test versus the shNC control. **f** Annexin-V and propidium iodide staining for apoptosis are quantified in the indicated stable cells. Data are means ± s.e.m. *p* value was calculated by student’s *t*-test versus the shNC control. **g** The indicated stable NCI-H1975 cells were injected into randomized athymic nude mice by tail-vein injection. Representative images of H&E-stained sections in the dissected lungs after inoculation for 6 weeks are shown, and the metastatic nodules were quantified based on the H&E-stained lung sections. Data are means ± s.e.m., *n* = 8, and the *p* values were calculated by student’s *t*-test. **h**, **i** The indicated stable cells were subjected to Western blotting. **j** The A549 stable cells were subjected to migration and invasion assays. The columns were mean of three independent experiments. Data are means ± s.e.m. *p* values were calculated by student’s *t*-test versus the shNC control. **k** The co-high and co-low subgroups of RMI2 and RUNX2 were determined based on the combination of both RMI2 and RUNX2 staining values. *n* = 93. Coefficient of correlation (r) and *p* value were calculated by the nonparametric Spearman’s test. **l** Kaplan–Meier survival analyses of the co-high and co-low subgroups of RMI2 and RUNX2. *p* = 0.0436 was calculated by the log-rank test. **m** A proposed model for the roles of RMI2 in lung cancer growth and metastasis

To investigate the functions of RMI2 in lung cancer, A549 and NCI-H1975 cells were utilized to generate stable cells with knockdown of RMI2, as RMI2 was higher in lung cancer cell lines compared to BEAS-2B, a lung normal bronchial epithelial cell line (Supplementary Fig. S2a, b). Cell viability and colony formation, as well as tumor growth in the xenograft mouse model, were decreased by knocking down RMI2 in both A549 and NCI-H1975 cells (Fig. 1c and Supplementary Fig. S2c–e). Such a function of RMI2 on tumor growth depends on BTR complex, as the inhibition on colony formation by knocking down endogenous RMI2 in these cells was completely rescued by re-introducing wild type RMI2, but not its mutants of RMI2-K24A and RMI2-W135A, which are well-known to abolish the interaction of RMI2 with RMI1, TOP3A and/or BLM^3^ (Fig. 1d). Consistently, γH2AX, a marker for DSBs, and apoptosis were substantially increased by knocking down RMI2 (Fig. 1e, f and Supplementary Fig. S2f, g). These results illustrate that RMI2 is crucial for lung cancer cell viability and tumor growth, which is dependent on BTR complex.

Interestingly, depletion of RMI2 also inhibited cell migration and invasion, whereas ectopic RMI2 increased the migration and invasion (Supplementary Fig. S3a, b). Moreover, using tail-vein injection mouse model, the number of lung metastatic nodules were decreased by knocking down RMI2 and increased by ectopic RMI2 in NCI-H1975 cells, respectively (Fig. 1g). Unexpectedly, RMI2 might regulate EMT, as E-cadherin was increased while SLUG was decreased by knocking down RMI2 in NCI-H1975 cells (Supplementary Fig. S3c). Then, we tested whether RMI2 affect RUNX2, as SLUG is a well-known downstream of runt-related transcription factor 2 (RUNX2),^4^ which plays a key role in migration and invasion in A549 cells (Supplementary Fig. S3d). Knockdown and ectopic RMI2 decreased and increased the protein level of RUNX2 in both A549 and NCI-H1975 cells, respectively (Fig. 1h and Supplementary Fig. S3e). The enhancements of migration and invasion by ectopic RMI2 were abolished by knocking down RUNX2 in cells (Fig. 1i, j), indicating that RMI2 regulates migration and invasion through up-regulating RUNX2. Supportively, RUNX2 by IHC was also higher in lung tumor tissues compared to normal lung tissues, and the high RUNX2 protein levels were significantly associated with poor outcomes in LUAD patients (Supplementary Figs. S3f–h).

To determine how RMI2 up-regulates RUNX2. First, ectopic of the RMI2 mutants of RMI2-K24A and W135A also increased the protein level of RUNX2 in both A549 and NCI-H1975 cells (Supplementary Fig. S4a), indicating that up-regulation of RUNX2 by RMI2 is independent of BTR complex. Second, the mRNA level of RUNX2 was not altered by either overexpression or knock-down of RMI2 in both A549 and NCI-H1975 cells (Supplementary Fig. S4b). Third, interaction between RMI2 and RUNX2 was detected when they were co-expressed in HEK-293T cells (Supplementary Fig. S4c); endogenous RUNX2 was easily immunoprecipitated by ectopic RMI2 in both A549 and NCI-H1975 cells (Supplementary Fig. S4d). Fourth, RMI2 was co-localized with RUNX2 in nuclei (Supplementary Fig. S4e); RUNX2 was distributed as multiple aggregates around nucleus by knocking down RMI2 in NCI-H1975 cells (Supplementary Fig. S4f), indicating that RUNX2 may be misfolded when RMI2 is knocked down in cells, as RUNX2 contains many disordered regions as predicted by d^2^p^2^ database (Supplementary Fig. S5). CHIP, a well-known E3 ligase, promotes the degradation of almost misfolded proteins including RUNX2^5^ through ubiquitin–proteasome pathway. Indeed, depletion of CHIP increased the protein level of RUNX2, while RUNX2 was decreased by ectopic CHIP in both A549 and NCI-H1975 cells (Supplementary Fig. S6a, b). Furthermore, RMI2, RUNX2, HSP70, HSP90, and CHIP could form a complex at their endogenous levels in NCI-H1975 cells (Supplementary Fig. S6c). However, CHIP could not be detected in the Flag-RMI2/RUNX2 complex in NCI-H1975 cells (Supplementary Fig. S6d), suggesting that the scarcely misfolded RUNX2 may exist when RMI2 is overexpressed in cells, and ubiquitination of RUNX2 mediated by CHIP was decreased by RMI2 (Supplementary Fig. S6e). Furthermore, the decrease of RUNX2 by knocking down RMI2 was significantly rescued with proteasome inhibitor bortezomib and lysosomal inhibitor Bafilomycin A1 (Supplementary Fig. 6f), as the aggregated proteins are generally degraded by proteosome and/or lysosome. These results determine that RMI2 may act as a chaperon to stabilize RUNX2 by facilitating its fold, which is independent of BTR complex.

There was a strong positive correlation between RMI2 and RUNX2 at their protein levels using 39 fresh-frozen human LUAD tissues (Supplementary Fig. S6a, b), which was further validated by IHC using a tumor tissue microarray (Fig. 1k and Supplementary Fig. S6c). The Kaplan–Meier survival analysis showed that lung cancer patients with low and high levels of both RMI2 and RUNX2 predicted better and poorer survivals, respectively (Fig. 1l). These results illustrate that elevated RMI2 is correlated with high RUNX2 in lung cancer.

In summary, we provide evidences for the first time that RMI2 is critical for growth and metastasis of lung cancer, which is dependent- and -independent of BTR complex, respectively, indicating that RMI2 may be a promising therapeutic target for lung cancer. As illustrated in Fig. 1m, synthesized in cytoplasm, RMI2 can be shuttled to nucleus, where it acts as BTR complex to maintain genomic integrity, which is required for cell viability and tumor growth. On the other hand, RMI2 may function as a chaperon molecule, which is independent of the BTR complex, to facilitate the fold of RUNX2, which in turn to avoid the degradation of RUNX2 by CHIP, consequently, SLUG is transcriptionally up-regulated by more RUNX2 in the nucleus to promote cancer metastasis.

## Supplementary information

Supplementary material
