# Supplementary material for: RMI2 plays crucial roles in growth and metastasis of lung cancer
Source: Signal Transduct Target Ther. 2020 Sep 3;5:188. doi: 10.1038/s41392-020-00295-4 (PMC7471275; doi:10.1038/s41392-020-00295-4)
Supplement: Supplementary file 1 — Supplementary material [file 41392_2020_295_MOESM1_ESM.docx]

Supplementary Materials for

**RMI2 plays crucial roles in growth and metastasis of lung cancer**

Weixiang Zhan^1,2^, Yina Liu^1^, Ying Gao^1^, Run Gong^1^, Wen Wang^3^, Ruhua Zhang^1^, Yuanzhong Wu^1^, Tiebang Kang^1^* and Denghui Wei^1^*

Correspondence to:

*Denghui Wei (Email: [weidh@sysucc.org.cn](mailto:weidh@sysucc.org.cn))

*Tiebang Kang (Email: [kangtb@sysucc.org.cn](mailto:kangtb@sysucc.org.cn))

**This PDF file includes:**

Materials And Methods

Figures S1 to S6

Tables S1 to S2

**MATERIALS AND METHODS**

Cell lines and culture

All cell lines used in this study were originally obtained from the ATCC as follows: HEK-293T, A549, NCI-H1975, NCI-H460, Beas-2B, NCI-H292, PC9, HCC827. All cells were incubated in humidified air at 37 °C with 5% CO2. The cell lines were cultured in DMEM (Gibco) with 10% foetal bovine serum (FBS), 100 U ml^-1^ of penicillin and 100 μg ml^-1^ of streptomycin. All cell lines used in this study were regularly authenticated by the STR method and have not been in culture for more than 2 months.

Plasmids

The cDNA of RMI2 were cloned by PCR from the cDNA of NCI-H1975 cells and inserted into pSin-EF2-puro-oligo backbone between BstBI and NheI. Myc-tagged CHIP, mcherry-RUNX2, flag-RMI2 and EGFP-RMI2 were also cloned into the pSIN-EF2-puro vector.

The shRNA expression constructs were in the pLKO.1-puro backbone. The sequences of the shRNAs used in this study were provided in the Supplementary Materials (Table S1).

Antibodies and reagents

The following antibodies were used for Western blotting: Flag rabbit antibody (1:2000; Cell Signaling; 14793), HA rabbit antibody (1:2000; Cell Signaling; 3724), GAPDH rabbit antibody (1:1000; Proteintech; 10494-1-AP), RMI2 rabbit antibody (1:1000; Abcam; ab122685), RUNX2 rabbit antibody (1:1000; Cell Signaling; 8486), SLUG rabbit antibody (1:1000; Cell Signaling; 9585), ECAD rabbit antibody (1:1000; Cell Signaling; 3195), NCAD rabbit antibody (1:1000; Cell Signaling; 13116), TWIST1 rabbit antibody (1:1000; Cell Signaling; 46702), CHIP rabbit antibody (1:1000; Bethyl; A310-572A), HSP70 mouse antibody (1:5000; Santa Cruz; sc-24), HSP90 mouse antibody (1:2000; Santa Cruz; sc-69703). The following reagents were used for cell: Bortezomib (Selleck; S1013) and Bafilomycin A1 (Selleck; S1413).

RNA extraction and qRT-PCR

The total RNA was extracted using the RNAprep Pure Cell/Bacteria Kit (Tiangen; DP430). The total RNA (1 μg) was reverse-transcribed using a HiScript II Q RT SuperMix for qPCR (Vazyme; R223-01). qRT-PCR was performed using the ChamQ Universal SYBR qPCR Master Mix (Vazyme; Q711-02) on LightCycler 480 (Roche). All qRT-PCR samples were repeated at least three times. The primers used for amplifying RMI2, RUNX2 and GAPDH were provided in the Supplementary Materials (Table S2).

Western blotting and immunoprecipitation

Cells were harvested and lysed in RIPA buffer (50 mM Tris-HCl pH 7.5, 150 mM NaCl, 1 mM EDTA, 1% NP40) containing Protease Inhibitors Cocktails set I (Calbiochem; 539131) and Phosphatase Inhibitor Cocktails set II (Calbiochem; 524625), and centrifuged at 12,000 rpm/min for 20 minutes at 4 °C. For immunoprecipitation, 20 μL of the beads was added and incubated with the lysates overnight at 4 °C. For endogenous IP, 1,000 μg of lysate was incubated with the indicated antibody (2 μg) overnight at 4 °C with protein A agarose beads. The beads were washed five times with RIPA buffer. The IPs and cell lysates were then boiled in gel loading buffer for 10 min and resolved by 12% SDS-PAGE. The proteins were transferred to Immobilon-P PVDF membranes (Millipore), which were then blocked in PBS with 5% nonfat milk and 0.1% Tween-20 and immunoblotted with primary antibodies overnight at 4 °C. Horseradish peroxidase-conjugated secondary antibody were used, and high-signal ECL substrate (Tanon) was used for detection.

Immunofluorescence

Cells were seeded into glass-bottomed culture dishes (NEST Biotechnology; 801002) one day before experiments. All transfection experiments were performed using Lipofectamine 2000 (Invitrogen). Cells were fixed with 4% paraformaldehyde for 15 min, permeabilized with 0.5% Triton X-100 for 15 min and blocked with 3% bovine serum albumin for 30 min at room temperature, and rinsed twice with PBS between interval step. Next, the cells were incubated with the primary antibodies for 2 h at room temperature or overnight at 4 °C. After rinsing three times with PBS, the cells were incubated for 2 h at room temperature with the following secondary antibodies: anti-mouse Alexa Fluor-594, anti-rabbit Alexa Fluor-488 (Molecular Probes, Invitrogen). Nuclei were stained with Hoechst 33342 (Molecular Probes, Invitrogen) for 2 min. The cells were imaged using laser scanning confocal microscopes (ZEISS, LSM880, ZEN2.6, 63× oil lens).

MTT assay

A 3-(4, 5-Dimethylthiazol-2-yl)-2, 5-diphenyltetrazolium bromide (MTT; Sigma-Aldrich, USA assay) was used to measure cell viability. Briefly, A549 and NCI-H1975 were seeded at a density of 3,000 cells per well in a 96-well microplate. The cells were incubated with 20 µL MTT reagent (5 mg/mL) for 4 hours, and then 150 µL DMSO was added to dissolve the formazan product and the MTT absorbance was detected at optical density (OD) 490 nm with the microplate reader once per day for 5 days. Experiments were performed three times. The tuplicate results of one representative experiment are shown.

Colony formation assay

Briefly, A549 and NCI-H1975 cells were plated into 6-well plate at 500 cells per well in triplicates and cultured for 12 days. Then, the cells were washed twice with PBS and fixed in ethanol for approximately 30 min and stained with 1% methyl violet in PBS for 60 min. Then, the methyl violet was washed out, and the numbers of colonies were counted.

Apoptosis assay

A549 and NCI-H1975 cells stably knocking down RMI2 were collected and stained with annexin V-FITC and propidium iodide (KeyGen, KGA-108). Stained cells were then examined with flow cytometry and results analysed with FlowJo. A representative result of three independent experiments is shown.

Migration and invasion assays

The cell migration and invasion assays were performed using chambers (Falcon

353504) containing polyethylene terephthalate membranes of 8 μm pore size. 5×10^4^ cells were suspended in serum-free medium and added to the upper chamber, which was coated with (invasion assay) or without (migration assay) the matrigel mix. And culture medium with 20% fetal bovine serum was placed into the lower chamber. After 12 h of incubation, the migrated or invaded cells were fixed, stained, and counted from five random fields under 10× objective lens using an inverted microscope (Olympus, Japan). All experiments were performed in duplicate and repeated three times.

Animal experiments

Briefly, 1×10^6^ A549 and NCI-H1975 cells stably expressing shNC or shRMI2 were subcutaneously injected into the left dorsal part of randomized 5-week-old male athymic nude mice. Tumors were measured twice weekly with callipers and the tumor volumes were calculated using the formula π/6×(width^2^×length). Mice were sacrificed when tumors reached 15 mm at the largest diameter and tumors were dissected. For the lung metastatic model, 1×10^6^ NCI-H1975 cells stably expressing shNC or shRMI2 and vector or overexpressed RMI2 were injected into the tail veins. All mice were sacrificed 6 weeks after injection, and the lungs were harvested. The metastatic nodules in each lung were counted.

Animal care and experiments were performed in strict accordance with the "Guide for the Care and Use of Laboratory Animals" and the "Principles for the Utilization and Care of Vertebrate Animals" and were approved by the Animal Research Committee of Sun Yat-sen University Cancer Center.

Human tissue specimens

Thirty-nine fresh-frozen human lung adenocarcinoma tissues were clinically and histopathologically diagnosed at the Sun Yat-sen University Cancer Center from December 2016 to September 2017 and were analysed in accordance with the ethical standards laid down in the Declaration of Helsinki. The fresh-frozen tissues were cut into pieces and homogenized in RIPA Lysis Buffer (Beyotime, P0013B) on ice for 30 min. The lysates were clarified by centrifugation at 12,000 g for 30 min at 4 °C and were stored at -80 °C for further use, including Western blotting analyses.

LUAD tissue array and immunohistochemistry

Human lung tumor tissue microarrays were purchased from Shanghai Outdo Biotech, China. The survival time was calculated from the day of operation to the end of the follow-up day or date of death because of recurrence and metastasis. RMI2 rabbit antibody (1:400; abcam; ab122685) and RUNX2 rabbit antibody (1:200; abcam; ab192256) were used for immunostaining. To score a tumour cell as positive, both nuclear and cytoplasmic staining were counted. For quantitative analysis, a histochemistry score (H-score) was calculated based on the staining intensity and percentage of stained cells using the Inform Tissue Finder system. The intensity score was defined as follows: 0, no appreciable staining in cells; 1, weak staining in cells comparable with stromal cells; 2, intermediate staining; 3, strong staining. The fraction of positive cells was scored as 0%-100%. The H-score was calculated by multiplying the intensity score and fraction score using the following formula:

[1×(% cells 1+) + 2×(% cells 2+) + 3×(% cells 3+)]

with a total range of 0-300. Tissue sections were examined and scored separately by two independent investigators blinded to the clinicopathologic data.

Statistical analyses

Statistical analyses were performed using Prism 8 (GraphPad) software, and data are generally expressed as the mean ± standard. Quantification analyses of tissue specimens were analysed using ImageJ software and were assessed by the Gaussian Population Pearson's test. Spearman’s rank correlation analysis was performed to evaluate correlations in gene expression in the tissue array. Other data were compared by Student’s *t*-test or the Mann-Whitney test. A *p* value less than 0.05 was considered

statistically significant.

Data availability

Gene-expression profiling interactive analysis in different cancer types and corresponding normal tissues was deposited at GEPIA based on TCGA and GTEx database (<http://gepia.cancer-pku.cn/>)[^1^](#_ENREF_1). Survival durations in different kinds of cancer with low and high expression of RMI2 compared was deposited at the Kaplan-Meier plotter based on TCGA (<http://kmplot.com/>). The prediction disordered regions of RUNX2 from d2p2 database of disordered protein predictions (<http://d2p2.pro/>)[^2^](#_ENREF_2).

**AUTHOR CONTRIBUTIONS**

W.Z., D.W., and T.K. conceptualization; W.Z., Y.L., and Y.G. data curation; W.Z., Y.L., Y.G., R.G., and W.W. data collection and analysis; W.Z., Y.L., Y.G., Y.W., and R.Z. methodology; W.Z., D.W., and T.K. writing; and D.W. and T.K. project administration.

**REFERENCE:**

1 Tang, Z. et al. GEPIA: a web server for cancer and normal gene expression profiling and interactive analyses. *Nucleic acids research* **45**, W98-W102, (2017).

2 Oates, M. E. et al. D²P²: database of disordered protein predictions. *Nucleic acids research* **41**, D508-D516, (2013).


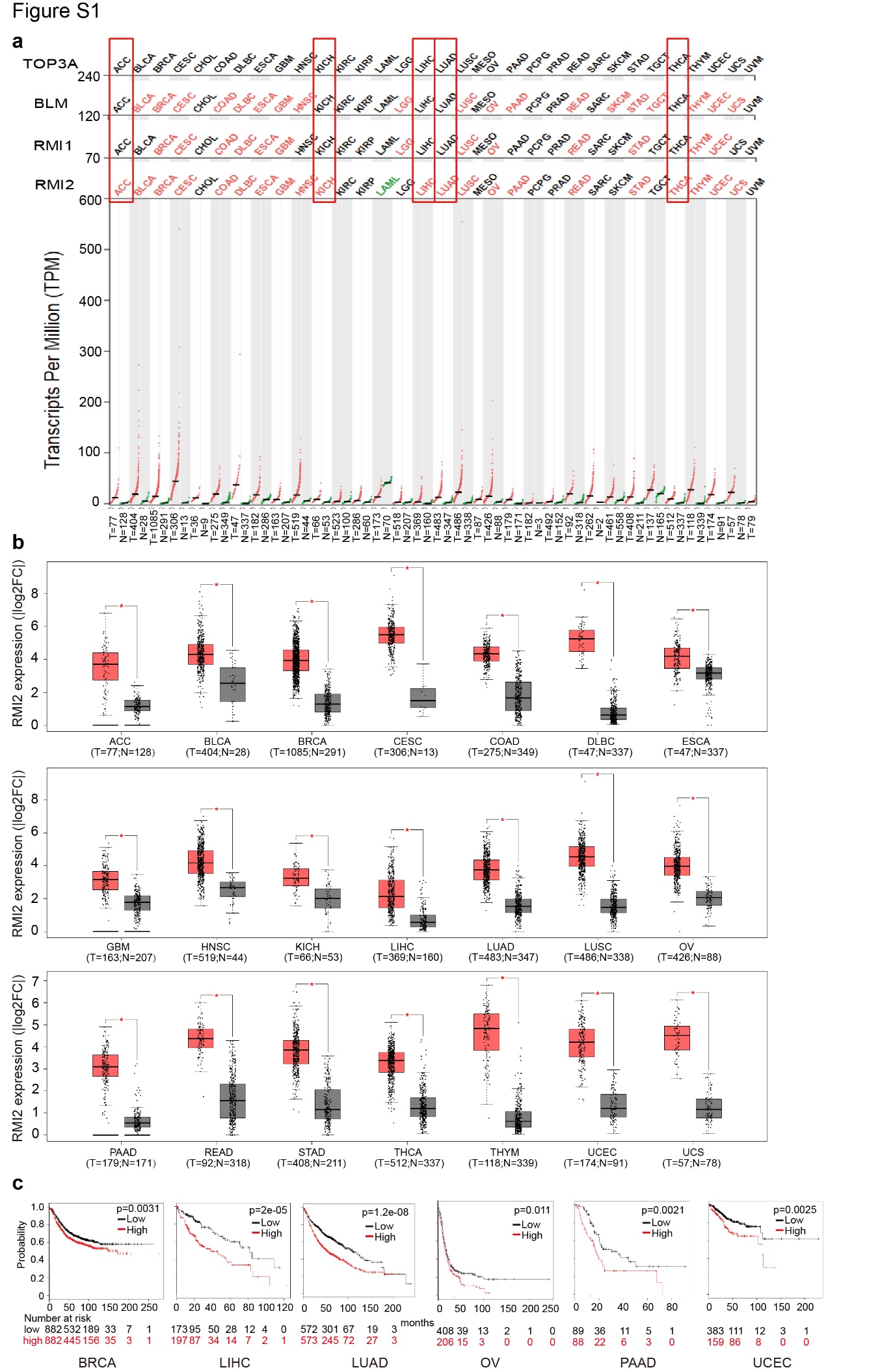


**Fig. S1 Elevated RMI2 was observed in multiple cancer types and was correlated with poor prognosis in cancer patients. a** The comparison of mRNA levels of TOP3A, BLM, RMI1 and RMI2 between cancer and noncancerous tissue was individually shown. The red and green fonts present the mRNA at significantly higher and lower in cancers than noncancerous tissues, respectively, while the black fonts present no difference. **b** mRNA levels of RMI2 were higher in multiple cancer types (red column) compared to their normal tissue (grey column) based on TCGA and GTEx databases. Student’s *t*-test; * *p*< 0.05. **c** Overall survival probabilities were generated based on the mRNA levels of RMI2 in the different cancer tissues. *p* values were shown using Kaplan-Meier plots and compared with the two-tailed log-rank test.

ACC, Adrenocortical carcinoma; BLCA, Bladder Urothelial Carcinoma; BRCA, Breast invasive carcinoma; CESC, Cervical squamous cell carcinoma and endocervical adenocarcinoma; CHOL, Cholangiocarcinoma; COAD, Colon adenocarcinoma; DLBC, Lymphoid Neoplasm Diffuse Large B-cell Lymphoma; ESCA, Esophageal carcinoma; GBM, Glioblastoma multiforme; HNSC, Head and Neck squamous cell carcinoma; KICH, Kidney Chromophobe; KIRC, Kidney renal clear cell carcinoma; KIRP, Kidney renal papillary cell carcinoma; LAML, Acute Myeloid Leukemia; LGG, Brain Lower Grade Glioma; LIHC, Liver hepatocellular carcinoma; LUAD, Lung adenocarcinoma; LUSC, Lung squamous cell carcinoma; MESO, Mesothelioma; OV, Ovarian serous cystadenocarcinoma; PAAD, Pancreatic adenocarcinoma; PCPG, Pheochromocytoma and Paraganglioma; PRAD, Prostate adenocarcinoma; READ, Rectum adenocarcinoma; SARC, Sarcoma; SKCM, Skin Cutaneous Melanoma; STAD, Stomach adenocarcinoma; TGCT, Testicular Germ Cell Tumors; THCA, Thyroid carcinoma; THYM, Thymoma; UCEC, Uterine Corpus Endometrial Carcinoma; UCS, Uterine Carcinosarcoma; UVM, Uveal Melanoma.


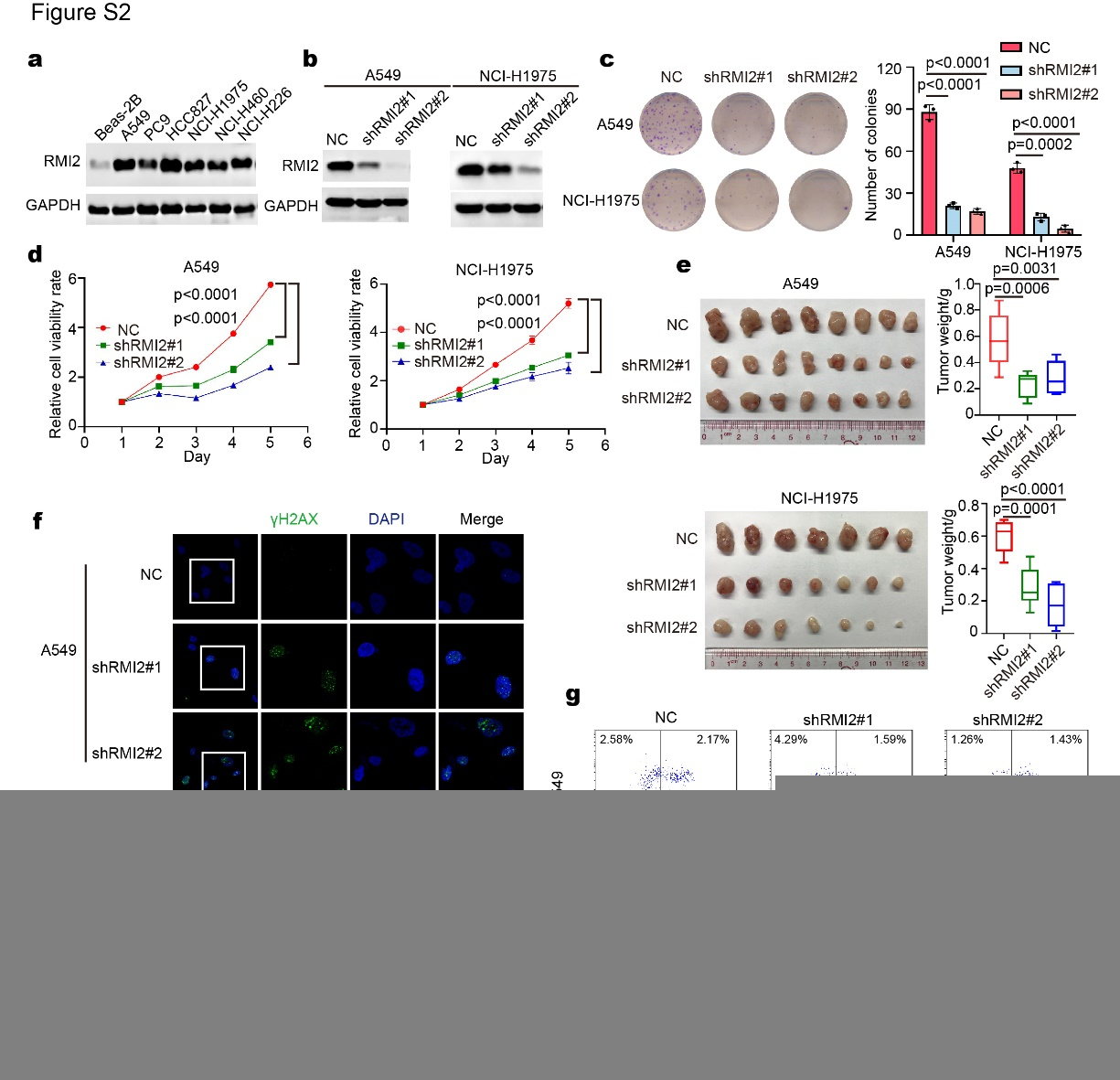


**Fig. S2** **RMI2 is crucial for lung cancer cell viability and tumor growth, which is dependent on BTR complex**. **a, b,** The indicated cell lines were analyzed by Western blotting. **c** The indicated stable cells were subjected to colony formation assay. The bars indicate the s.e.m. The results are expressed as the mean ± s.e.m. (n=3). *p* values were calculated by student’s *t*-test. **d** Cell viability of the indicated stable cells was measured by MTT assay. n=6. *p* values were calculated by Two-way ANOVA. **e** The indicated stable cells were xenografted subcutaneously on the flank of nude mice (n=8/group). Tumors were dissected from nude mice and weighed. Data are means ± s.e.m. of tumor weight. *p* values were calculated by Two-tailed student’s *t*-test versus the shNC control. bars indicate the SEM. **f** Representative γH2AX immunofluorescence images are shown in the indicated stable cells. **g** Representative Annexin-V and propidium iodide staining for apoptosis are shown.


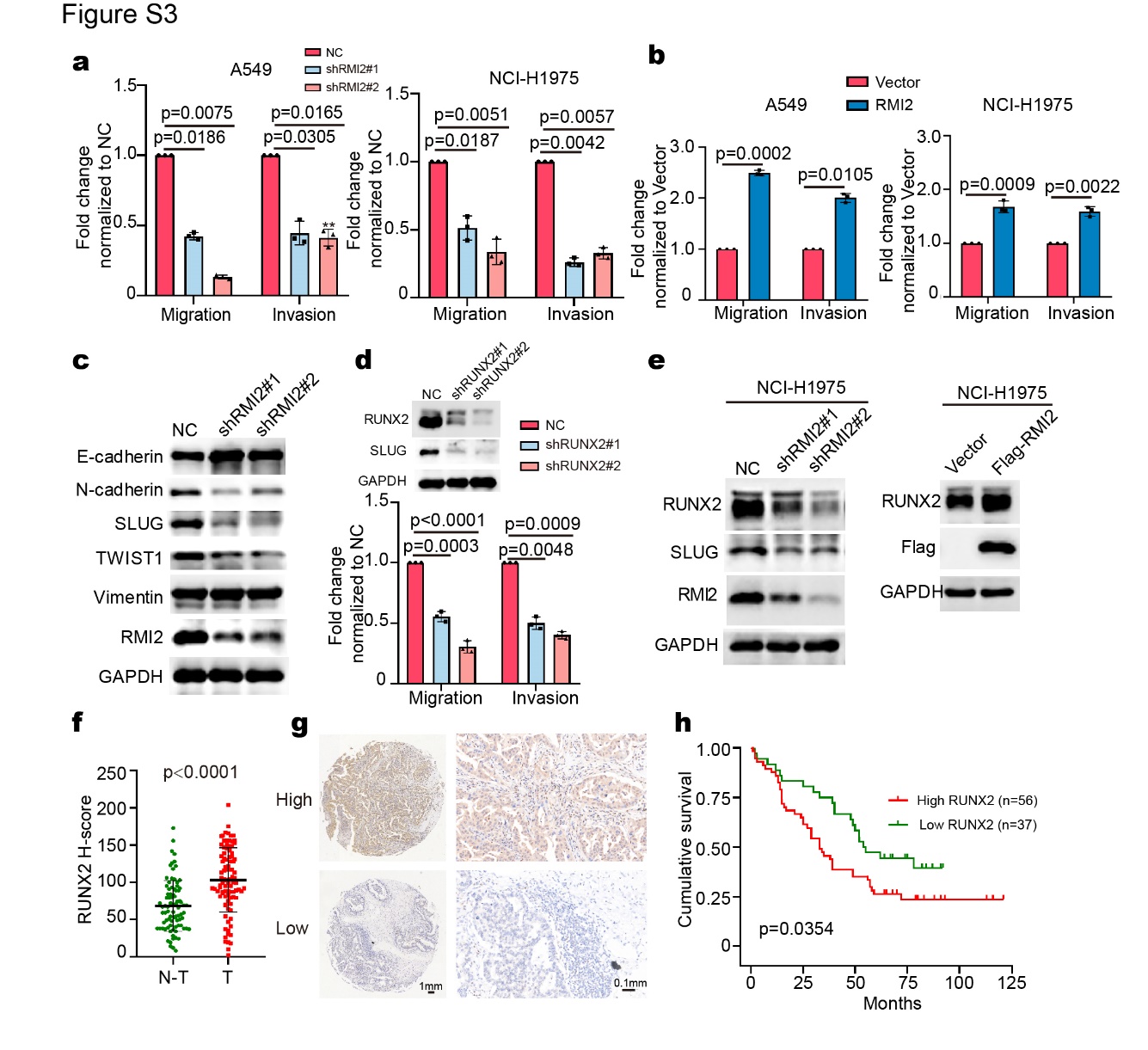


**Fig. S3** **RMI2 promotes migration and invasion of lung cancer cells.** **a, b** The indicated stable cells were subjected to migration and invasion assays. The columns were mean of three independent experiments. Data are means ± s.e.m. *p* values were calculated by student’s *t*-test versus the shNC control. **c, e** The indicated stable cells were subjected to Western blotting. **f** IHC staining of the primary human LUAD tissue microarray and adjacent noncancerous tissues. Scatter plot graph showing a statistical analysis of RUNX2 expression in LUAD and adjacent noncancerous tissues. Data are means ± s.e.m. *p*<0.0001 by student’s *t*-test. **g** Representative immunohistochemical staining images of RUNX2 in LUAD tissues used in **f. h** Overall survival curves were generated based on the protein levels of RUNX2 in LUAD tissue microarray. *p*=0.0354 using Kaplan-Meier plots and compared with the log-rank test.


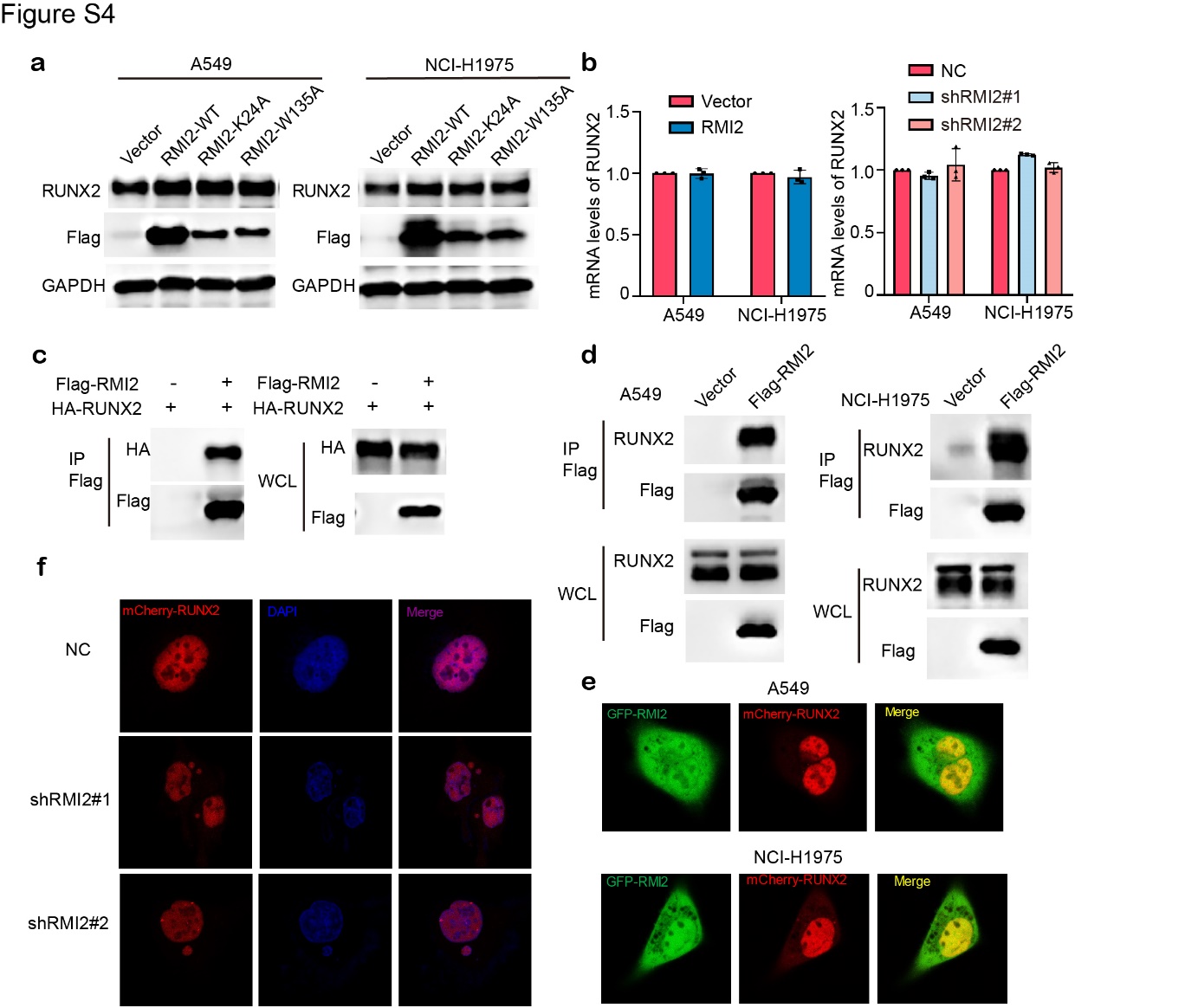


**Fig. S4** **RMI2 acts as a chaperon to stabilize RUNX2 by facilitating its fold, which is independent of BTR complex. a** The indicated stable cells were subjected to Western blotting. **b** The mRNA level of RUNX2 was not changed at both depletion and overexpression of RMI2 in both A549 and NCI-H1975 cells. **c, d** Western blotting analyses of whole-cell lysates (WCL) and immunoprecipitation (IP) from HEK-293T cells **(c)** or A549 or NCI-H1975 cells **(d)** using anti-flag antibody. **e** Immunofluorescence of GFP-RMI2 (green) with mCherry-RUNX2 (red) in the indicated stable A549 and NCI-H1975 cells. **f** Immunofluorescence of mCherry-RUNX2 (red) and DAPI (blue) in the indicated stable NCI-H1975 cells.


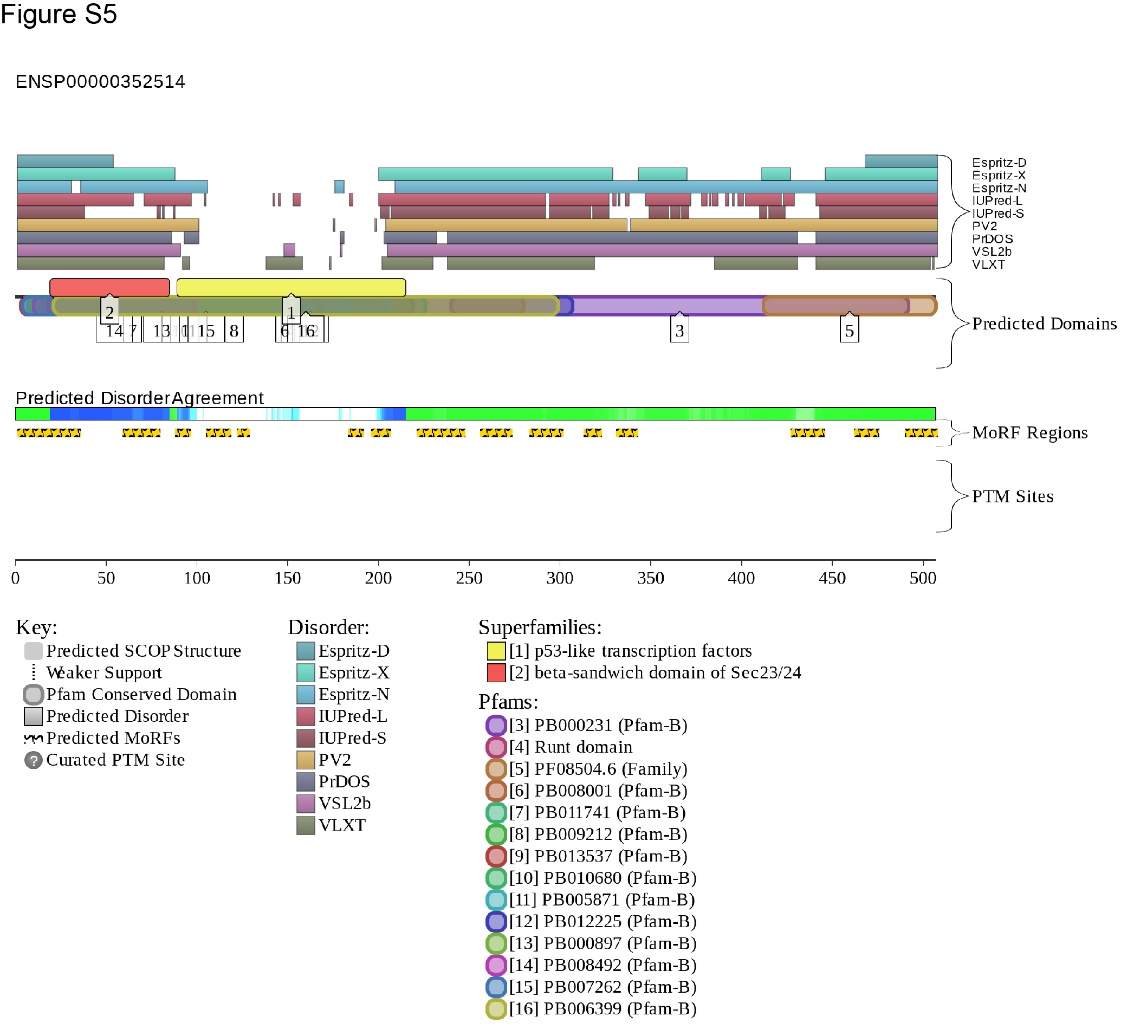


**Fig. S5 The prediction disordered regions of RUNX2 from d^2^p^2^ database.** RUNX2 has widely disordered regions as predicted, as mentioned in Methods.


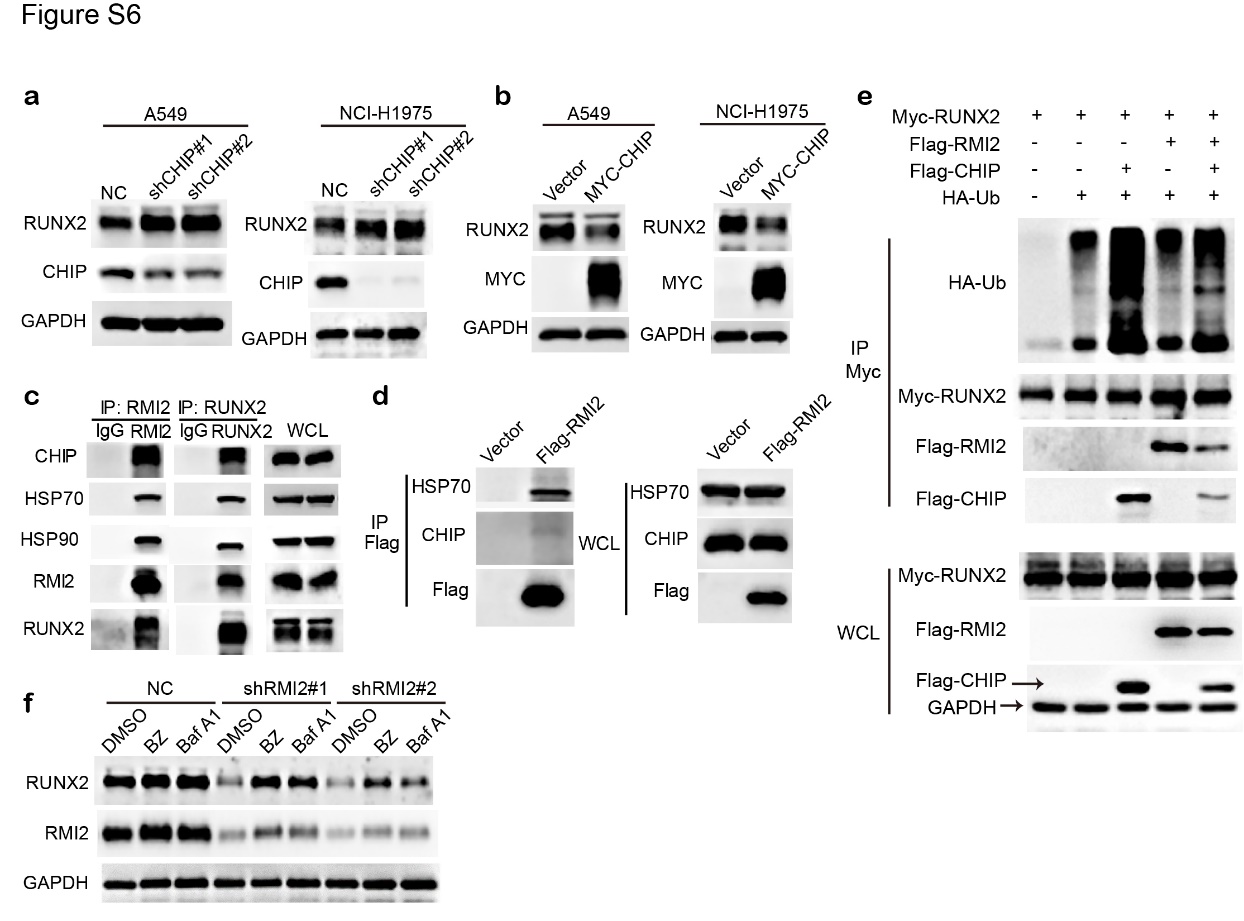


**Fig. S6 Misfolding RUNX2 is degraded by CHIP. a, b** The indicated stable A549 and NCI-H1975 cells were subjected to Western blotting.**c** Western blotting analyses of whole-cell lysates (WCL) and immunoprecipitation (IP) from NCI-H1975 cells using anti-RMI2 or anti-RUNX2 antibodies. **d** Western blotting analyses of whole-cell lysates (WCL) and immunoprecipitation (IP) from NCI-H1975 cells using anti-flag antibody. **e** Western blotting analyses of whole-cell lysates (WCL) and immunoprecipitation (IP) from HEK-293T cells using anti-myc antibody. **f** The indicated stable A549 cells were treated with DMSO, 1 μM of the proteasome inhibitor bortezomib (BZ) and 400 nM of the lysosomal inhibitor Bafilomycin A1 (Baf A1) for 6 hrs, and then were subjected to Western blotting.


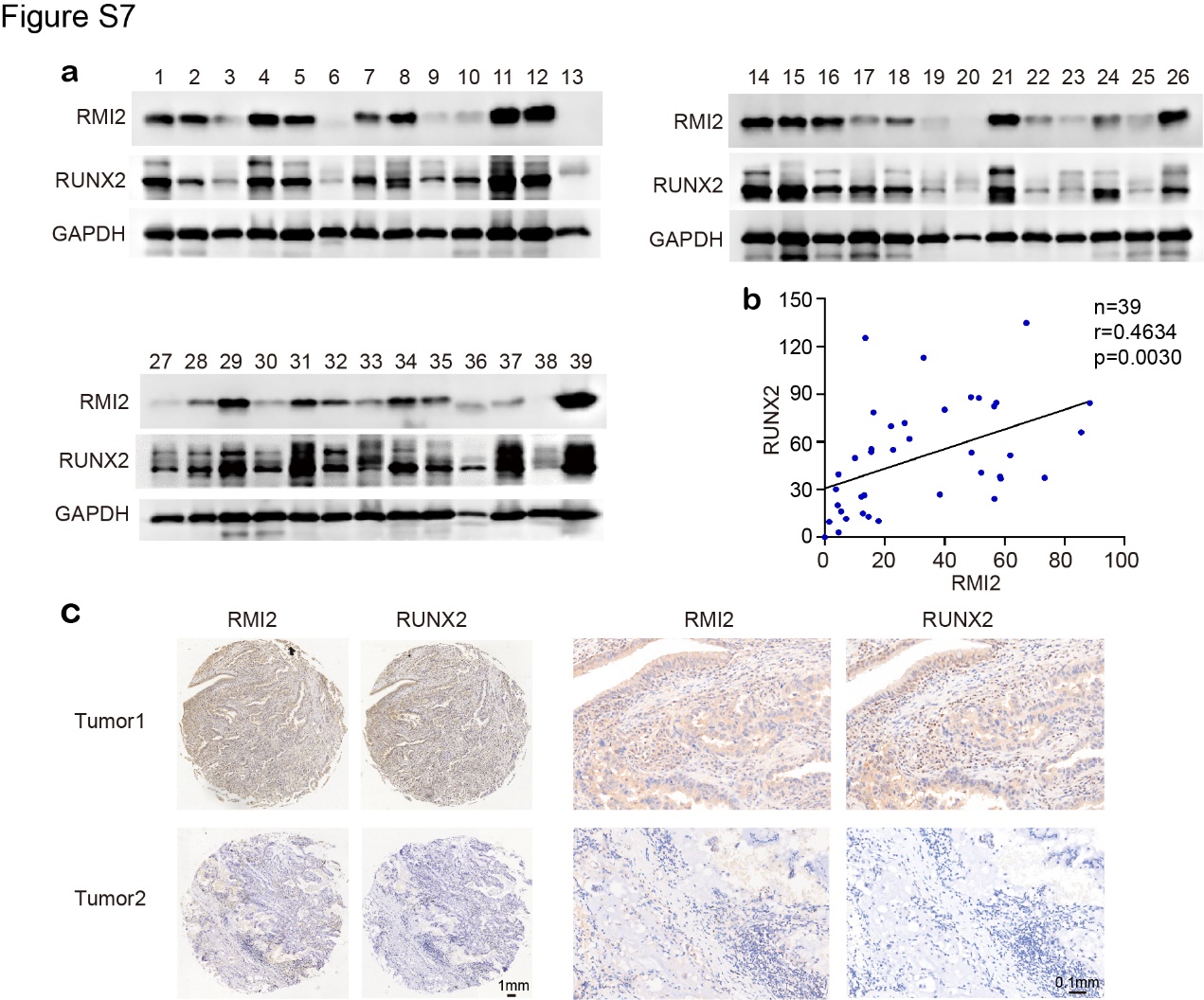


**Fig. S7 Elevated RMI2 is correlated with high RUNX2 in lung cancer**. **a** Western blotting analyses of the protein levels of both RMI2 and RUNX2 in freshly frozen human LUAD tissues. **b** The correlation scatter plots are shown based on the protein levels of RMI2 and RUNX2. n=39. Coefficient of correlation (r) and *p* value were calculated by the nonparametric Spearman’s test. **c,** Representative Images of human LUAD tissue microarray separately stained for RMI2 and RUNX2.

**Table S1. The sequences of shRNAs used in this study.**

| shRMI2-1 | 5’CCGGGCAGGAAGACAGACTGTGTAACTCGAGTTACACAGTCTGTCTTCCTGCTTTTTG-3’ |
| --- | --- |
| shRMI2-2 | 5’CCGGGCTTCGGACACCACTCAAACACTCGAGTGTTTGAGTGGTGTCCGAAGCTTTTTG-3’ |
| shRUNX2-1 | 5’CCGGCAAATTTGCCTAACCAGAATGCTCGAGCATTCTGGTTAGGCAAATTTGTTTTTG-3’ |
| shRUNX2-2 | 5’CCGGCAGCACTCCATATCTCTACTACTCGAGTAGTAGAGATATGGAGTGCTGTTTTTG-3’ |
| shCHIP-1 | 5’CCGGGAAGAGGAAGAAGCGAGACATCTCGAGATGTCTCGCTTCTTCCTCTTCTTTTTG-3’ |
| shCHIP-2-F | 5’CCGGCGCGAAGAAGAAGCGCTGGAACTCGAGTTCCAGCGCTTCTTCTTCGCGTTTTTG-3’ |

**Table S2. The sequences of quantitative real-time PCR primers.**

| RMI2-F | 5’-ATGCAGGGCAGGGTAGTGAT-3’ |
| --- | --- |
| RMI2-R | 5’-CTTTCCTGGGACTAGACAGGG-3’ |
| RUNX2-F | 5’-GATGCGTATTCCCGTAGATCC-3’ |
| RUNX2-R | 5’-CTGTTGCGCAGCCACCAC-3’ |
| GAPDH-F | 5’-TGACTTCAACAGCGACACCC-3’ |
| GAPDH-R | 5’-CTGGTGGTCCAGGGGTCTTA-3’ |
